# Effectiveness of laser welding in cerclage wiring fixation: a biomechanical study

**DOI:** 10.3389/fsurg.2023.1134986

**Published:** 2023-09-29

**Authors:** Nachapan Pengrung, Paphon Sa-ngasoongsong, Konlawat Sabsuantang, Nutchanat Thongchuea, Eakkachai Warinsiriruk

**Affiliations:** ^1^ Department of Orthopedics, Faculty of Medicine Ramathibodi Hospital, Mahidol University, Bangkok, Thailand; ^2^ Chakri Naruebodindra Medical Institute (CNMI), Faculty of Medicine Ramathibodi Hospital, Mahidol University, Samut Prakan, Thailand; ^3^ Department of Industrial Engineering, Faculty of Engineering, Mahidol University, Nakhon Pathom, Thailand

**Keywords:** laser welding, cerclage wiring fixation, single loop, double loop, tensile testing

## Abstract

**Introduction:**

Cerclage wiring is a common orthopedic procedure for fracture fixation. However, previous studies reported wiring-related perioperative complications, such as wire loosening or breakage, with an incidence rate of up to 77%. Recently, the use of laser welding on medical implants was introduced to connect biomedical materials. This study used laser technology to weld between wires after conventional cerclage fixation. We hypothesized that the laser welding could significantly increase the biomechanical properties of cerclage wiring fixation.

**Materials and methods:**

Twenty-five wiring models underwent biomechanical tests in five cerclage wiring configurations (five models per group), namely, (1) single loop, (2) single loop with laser welding, (3) double loop, (4) double loop with one-side laser welding, and (5) double loop with two-side laser welding. Characteristics such as load to failure, mode of failure, and wiring failure were compared between groups. The biocompatibility for a 316L stainless steel wire with laser welding was evaluated via an *in vitro* hemolysis test.

**Results:**

Mean load to failure of the double loop with one-side and two-side laser welding groups were 3,596 ± 346 N and 3,667 ± 240 N, which were significantly higher than for the double-loop group (2,919 ± 717 N) (*p* = 0.012 and *p* = 0.044, respectively). Conversely, no significant difference was shown in the comparison of the mean load to failure between the single loop and the single loop with laser-welded cerclage wire (1,251 ± 72 N, 1,352 ± 122 N, and *p* = 0.12). Untwisted wire and wire breakage were the most common mode of failure. All welding specimens revealed non-hemolytic effects from *in vitro* hemolysis test.

**Conclusion:**

Laser welding on cerclage wiring significantly increases the biomechanical property of double cerclage wire fixation. However, further biocompatibility tests and clinical studies are still recommended.

## 1. Introduction

Cerclage wiring fixation is a common orthopedic procedure, which was first proposed over 200 years ago (1), and nowadays remains popular in the fracture fixation procedure such as periprosthetic fracture, femoral fracture, or patellar fracture (2). In general, the cerclage wiring fixation was performed by using the standard stainless steel wire. However, regarding the cerclage materials, these options could be categorized into metallic materials (as stainless steel wire and cable) and non-metallic materials (as fiber tape and heavy suture). Recent studies showed that the newer cerclage materials have some advantages over the standard stainless steel wire. For example, Förch et al. reported the superior biomechanics of cable cerclage in addition to a plate osteosynthesis of distal tibia fracture (3). Hägerich et al. reported that the non-metallic cerclage provides similar stability to metal cerclage and reduces metallic risk profile (4). Nevertheless, the stainless steel wire remains the most common cerclage material because of surgeon familiarity, cost-effectiveness, and reliable biomechanical property.

Regarding the use of stainless steel wire in cerclage wiring fixation, previous studies also demonstrated many different surgical techniques, such as novel instruments, tightening methods, and wiring configurations that could be used for improving the fixation stability by stainless steel wire (5–9). Schultz et al. reported that two single-loop wires were stronger than a continuous double-loop wire (7). Lenz et al. reported that the double-looped technique provided better fixation stability compared to a single-looped technique (8). Wähnert reported that increasing the wire diameter and cerclage handling techniques—such as twisting under traction, cutting the wire outside the twist, and forward-bending the twist—affected the quality of a cerclage fixation (9). However, postoperative complications with fixation failure caused by wire breakage or loosening still occur in 2%–77% of cases (10). From the literature review, the two most common scenarios of fixation failure are the following: first, overtightened wire twisting creates plastic deformation at the initial turning point of the twisted wire to make the weakest point the cause of wire breakage (9, 10), and, second, inadequately tightened wire twisting causes untwisting or wire loosening (6, 7). Recently, laser welding technology had been used as a biomedical engineering tool due to the ability to join and seal the implanted biomedical materials, such as titanium alloys, cobalt–chromium alloys, stainless steel, and polymer-based materials (11). The laser beam was designed to deliver thermal energy to the focal point of different material surfaces, such as metal, and creating a fusion between them—and therefore—could be used for strengthening, repairing, or refurbishing medical devices and lead to the long-term durability of implants, especially in the dental procedure (11, 12). In 2020, we successfully developed the experimental protocol of using the laser welding for creating the fusion between the stainless steel cerclage wires. To the best of our knowledge, no previous study had investigated the biomechanical property of laser welding for cerclage wiring fixation in orthopedic surgery.

Therefore, this study aims to compare the biomechanical property between cerclage wire fixation with and without laser welding and evaluates the clinical safety with blood for the 316L stainless steel wire with laser welding via an *in vitro* hemolysis test. We hypothesized that laser welding is effective and safe for cerclage wire fixation.

## 2. Materials and methods

This study has been reviewed and approved by the Institutional Review Board at Mahidol University, based on the Declaration of Helsinki (COA. No. MURA2018/618, Protocol number 08-61-61).

### 2.1. Specimen preparation

**Figure 1:**
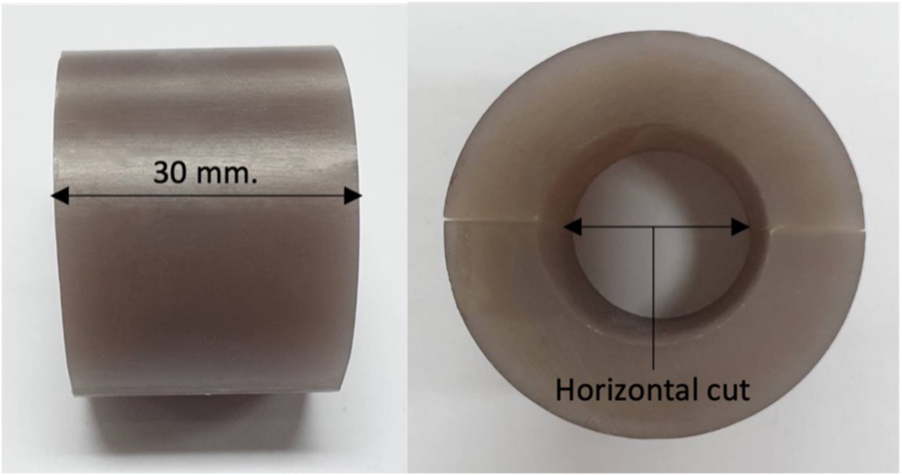
Synthetic bone preparation.

In this study, a synthetic bone [fourth generation (SKU: 3403-31), Sawbones, Vashon, WA, USA] with an external diameter of 40 mm and wall thickness of 8.9 mm was used. Twenty-five bone models were prepared using the same method, each model cut with a length of 30 mm; each model was then horizontally cut in half, as presented in Figure 1.

### 2.2. Study groups

**Figure 2:**
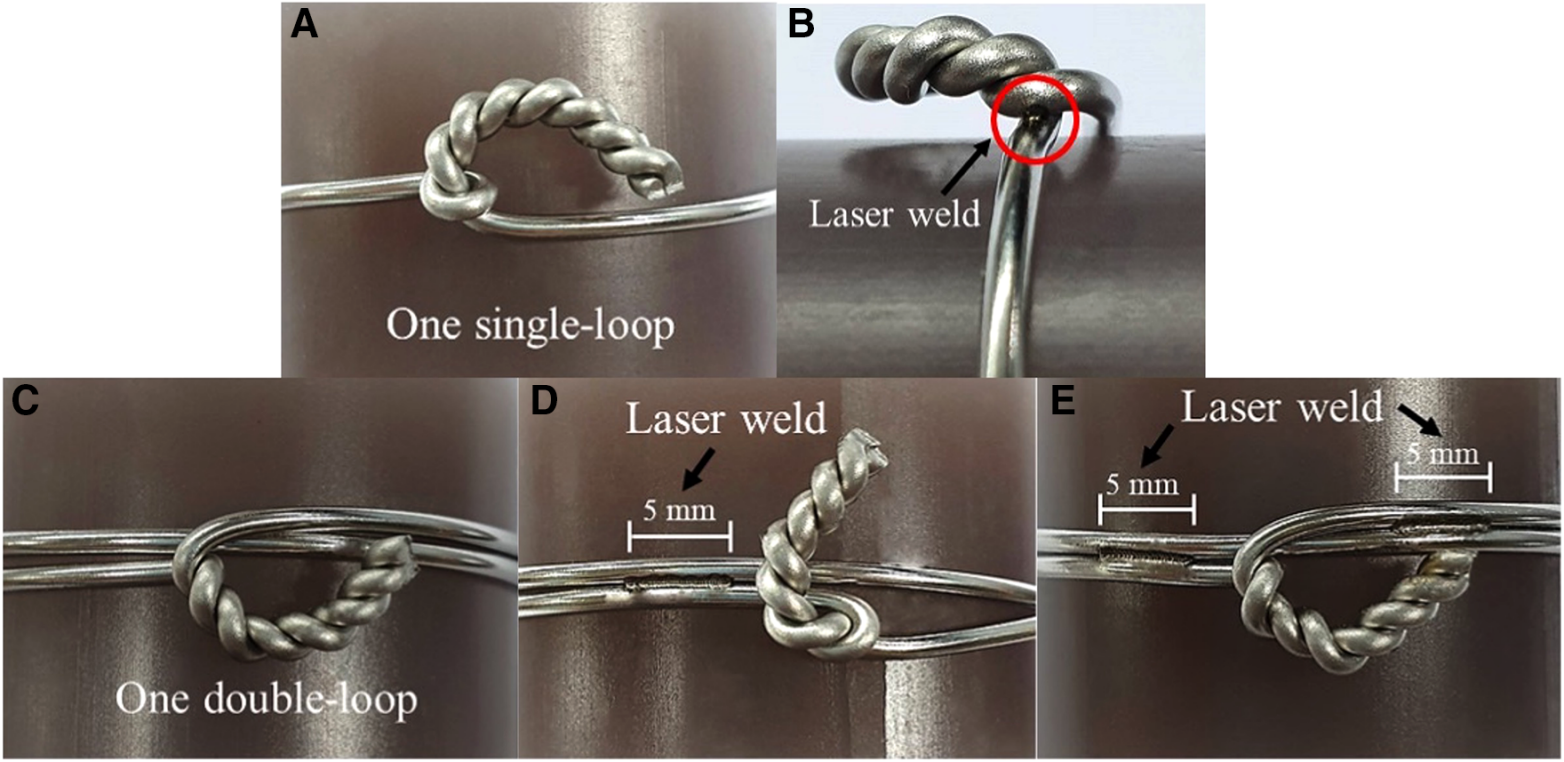
Cerclage wiring fixation method.

The 316L stainless steel wire (Synthes GmbH, Solothurn, Switzerland) that consists of 18% Cr, 13% Ni, 2.7% Mo, 1.7% Mn, and 0.3% C (13) with a diameter of 1.5 mm was used for the cerclage wiring techniques. The cerclage wiring was all prepared by one of the authors (NP), an orthopedic trauma expert, for five different cerclage configurations (five groups, Figure 2) with five models per cerclage configuration. In laser welding groups, the process was performed on the laser welding machine AHL-Laser (model XBW-400). The beam source is Nd:YAG (wavelength 1,064 nm). The laser welding parameters were set up using literature references from our previous study (Table 1) (14). Because of the tiny location for welding which limits the transfer of the energy from the laser to the wire in the single-loop group, we set a higher voltage and pulse width to create a higher heat input compared with the double-loop group:

(1) Single loop (Figure 2A).
(2) Single loop with laser welding (Figure 2B). Cerclage wire with one single loop was welded at the innermost turn of the twisted knot. The laser parameter for welding on the single loop was fixed at a charge voltage of 90 V, a pulse width of 2.5 ms, and a multi-frequency of 6 Hz (Table 1).
(3) Double loop (Figure 2C).
(4) Double loop with laser welding for one side (Figure 2D). Cerclage wire with one double loop was welded on wire to wire with 5 mm length of welding at one side of the twisted knot. The laser parameter for welding a double loop was fixed at a charge voltage of 80 V, a pulse width of 1.5 ms, and a multi-frequency of 6 Hz (Table 1).
5) Double loop with laser welding for two sides (Figure 2E). Cerclage wire with one double loop was welded on wire to wire with 5 mm length of welding at two sides across the twisted knot. The laser parameter for welding a double loop was fixed at a charge voltage of 80 V, a pulse width of 1.5 ms, and a multi-frequency of 6 Hz (Table 1).

**Table 1:** Laser parameters for the experiment.

Parameters	Level	
Cerclage wire	Single	Double
Charge voltage (V)	90	80
Pulse width (ms)	2.5	1.5
Multi-frequency (Hz)	6	
Ar gas shielding (L/min)	10	
Defocused length (mm)	56	
Beam diameter (mm)	1.76	
Length of welding (mm)	5	

### 2.3. Tensile testing

**Figure 3:**
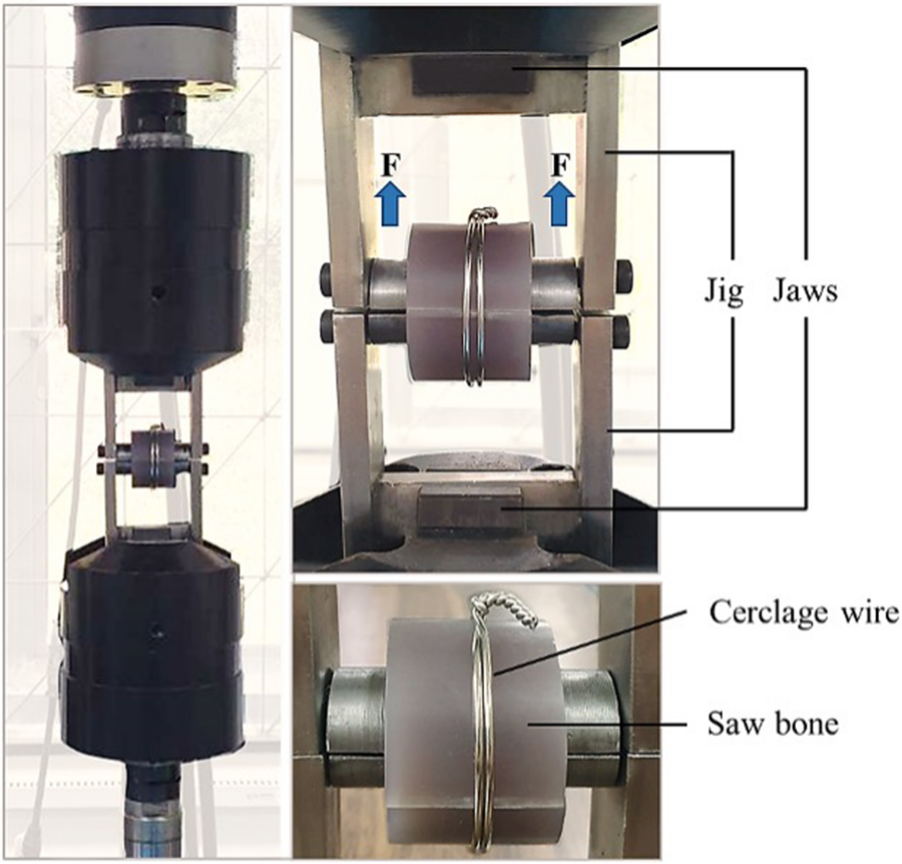
The tensile testing.

The experimental setup in this biomechanical study was performed using a servo-hydraulic testing machine (Jinan Testing Equipment IE Corporation) with a 20 kN load cell. The jig was designed as two metallic half cylinders with a radius of 10.6 mm forming a full cylinder with a cylinder bar length of 50 mm (Figure 3). The upper and lower half-cylinder parts were affixed with the testing machine, and the tensile load was applied to the specimens at a rate of 50 mm/min (15). All cerclage wiring specimens were tested until the wire breaks. The load of failure for the cerclage wire was measured.

### 2.4. *In vitro* hemolysis test

The biocompatibility with blood for the 316L stainless steel wire with laser welding was evaluated via an *in vitro* hemolysis test. The test was performed according to ASTM F756-13, ISO 10993-4, and ISO 10993-12 protocols (16). Three random specimens from low (80 V) to high (90 V) laser parameters were chosen for the test and then sterilized for 30 min by UV, and blood sampling was collected from at least three people. Human blood cells were treated with the calcium and magnesium-free phosphate-buffered saline (CMF-PBS) extracts to determine whether they caused an *in vitro* hemolysis effect. CMF-PBS and high-density polyethylene (HDPE) of 10 ml were used as the negative control, including positive controls using CMF-PBS and 18 MΩ of 10 ml. The human blood cells using these specimens were maintained in a tube for 3 h at 37 ± 2°C; each tube was gently inverted twice approximately every 30 min to maintain contact with the blood and material. Each supernatant was added by Drabkin's solution to define the hemoglobin concentration. The absorbance of the solution was measured with a spectrophotometer at a wavelength of 540 nm. According to the Standard Practices for Assessment of Hemolytic Properties of Materials, a hemolytic grade can be classified into three levels: hemolytic, defined as consisting of more than 5%; slightly hemolytic, defined as a percentage between 2% and 5%; and non-hemolytic, defined as a percentage between 0% and 2%.

### 2.5. Statistical analysis

Statistical analysis was performed using Minitab® 19 Statistical Software. The Kolmogorov–Smirnov test was used to determine the normality of the data. The data were presented as mean ± standard deviation and were used to compare the groups with ANOVA test and unpaired *t*-test. A *p*-value of <0.05 was considered statistically significant.

## 3. Results

**Figure 4:**
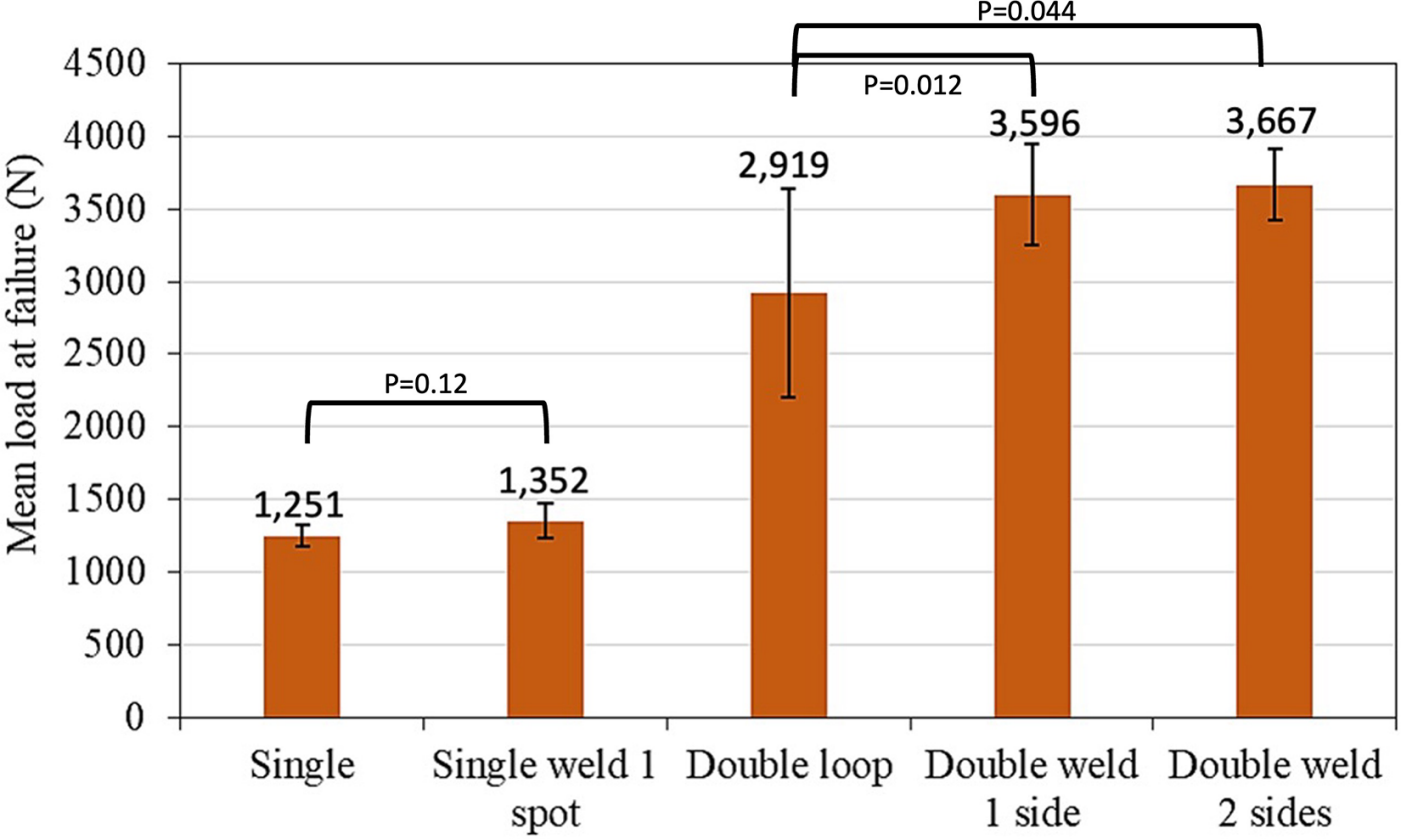
Mean load to failure (*N*).

Table 2 and Figure 4 present the result of the tensile test in five cerclage wiring groups and the mode of cerclage wire failure in each group. The mean load to failure was significantly different between the groups (*p* < 0.05). For the single-loop configurations, no significant difference was found between the conventional single-loop cerclage wire group and the single-loop cerclage wire with laser welding group (1,251 ± 72 N vs. 1,352 ± 122 N, *p* = 0.12). The average welding length in this single loop with the laser welding group is 0.74 mm. Meanwhile, among the double-loop cerclage wiring configurations, the double-loop cerclage wiring with both one-sided and two-sided laser welding groups (3,596 ± 346 N and 3,667 ± 240 N, respectively) had a significantly higher mean load to failure compared with the conventional double-loop cerclage wiring group (2,919 ± 717 N, *p* = 0.012 and 0.044, respectively).

**Table 2:** Mean load to failure and mode of failure at different wire fixation methods.

Study groups	Load to failure (*N* ± SD)	Mode of failure	
1. Single loop	1,251 ± 72	Untwist	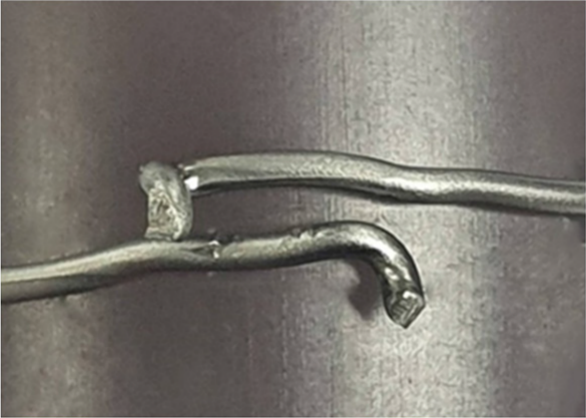
2. Laser welding on single loop	1,352 ± 122	Untwist	
3. Double loop	2,919 ± 717	Untwist	
4. Laser welding on double loop (weld one side)	3,596 ± 346	Wire breakage between twisted knot and welded area	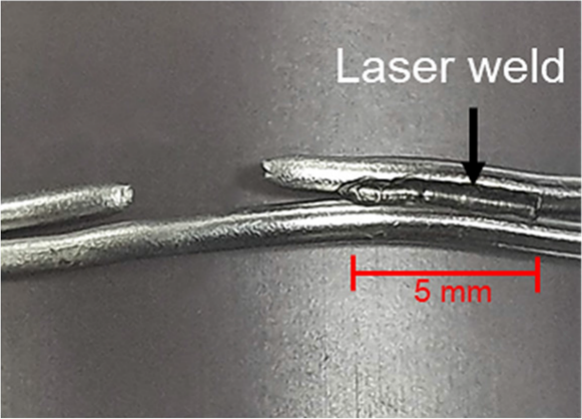
5. Laser welding on double loop (weld two sides)	3,667 ± 240	Wire breakage between twisted knot and welded area	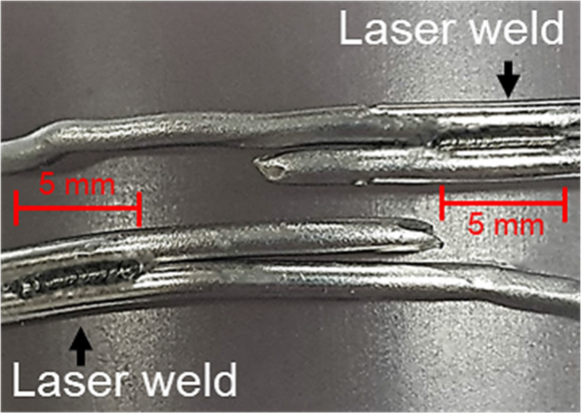

Regarding the mode of failure, the single-loop cerclage wiring with and without laser welding and double-loop cerclage wiring groups were failed by the untwisting of the knot. Conversely, the double-loop cerclage wiring with laser-welded groups failed via a wire breakage between the knot and welded area (Table 2). The average breakage location was 1.49 ± 1.19 mm (range 0.18–3.43 mm) in the laser welding for the one-sided group and 2.27 ± 0.75 mm (range 1.6–3.49 mm) and 5.36 ± 1.63 mm (range 3.82–7.73 mm) in laser welding for the two-sided group compared with the welded area (Table 3).

**Table 3:** Average breakage distance.

Study groups	Average breakage distance	
1. Laser welding on double loop (weld one side)	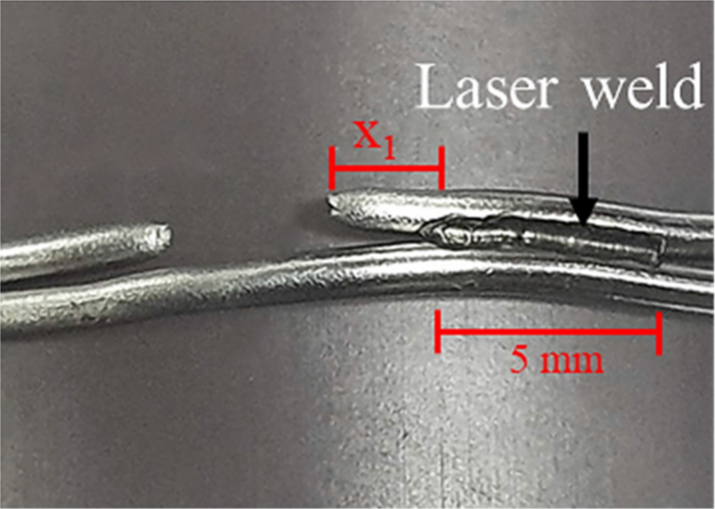	*X*_1_ = 1.49 ± 1.19 mm (range 0.18–3.43 mm)
2. Laser welding on double loop (weld two sides)	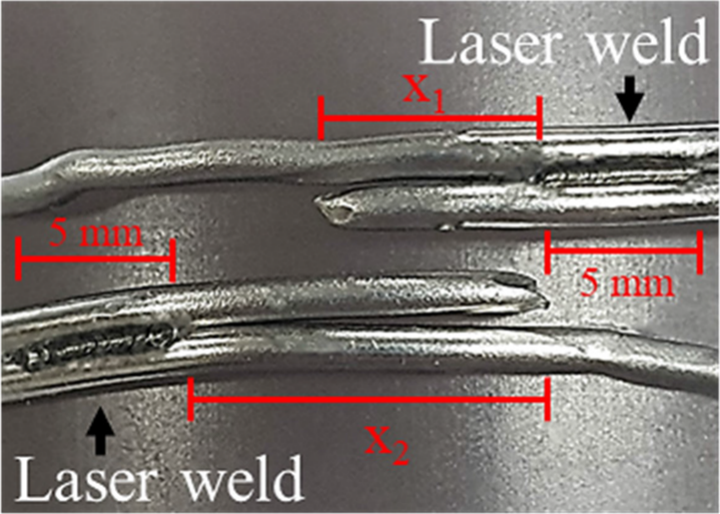	*X*_1_ = 2.27 ± 0.75 mm (range 1.6–3.49 mm) *X*_2_ = 5.36 ± 1.63 mm (range 3.82–7.73 mm)

The result of the hemolysis test is reported in Table 4. The mean percentage of hemolysis is below 2%, which is 0.355 (±0.11) at low laser parameters and 0.246 (±0.09) at high laser parameters. These results show that the hemolysis effect on the laser-welded surface of the 316L stainless steel wire is non-hemolytic (non-blood toxic), according to the Standard Practices for Assessment of Hemolytic Properties of Materials.

**Table 4:** Effect of hemolysis on 316l stainless steel with laser welding.

Sample	Hemoglobin of supernatant from extract (mg/ml)	% Hemolysis	Mean % hemolysis	SD	Hemolytic index	Hemolytic grade
Laser parameter: 80 V, 1.5 ms, 6 Hz						
Test item extract (A)—100%	0.003	0.266	0.355	0.107	0	Non-hemolytic
Test item extract (B)—100%	0.004	0.325				
Test item extract (C)—100%	0.006	0.473				
Laser parameter: 90 V, 2.5 ms, 6 Hz						
Test item extract (A)—100%	0.002	0.192	0.246	0.094	0	Non-hemolytic
Test item extract (B)—100%	0.004	0.355				
Test item extract (C)—100%	0.002	0.192				

## 4. Discussion

The current study aims to evaluate the biomechanical property of laser welding technology in a common orthopedic procedure, that is, cerclage wire fixation. Our results showed that the double-loop cerclage wiring with laser welding (either in one-sided or two-sided welded groups) provided significantly better biomechanical strength (load to failure under tensile traction) than conventional single- or double-loop cerclage wire fixation and single loop with laser welding (at the knot). However, no significant difference existed in the load to failure between double loop with one-sided welding and those with two-sided welding groups. Moreover, the results from this study showed that the mode of failure from double-loop cerclage wire fixation with laser welding differs from the single- or double-loop cerclage wire fixation without welding and the single loop with welding on the twisted knot. The failure mode for double loop with one-sided or two-sided welding groups was the breakage of the wire, while that for the other groups was untwisted wire. This difference could be explained by the laser welding potentially increasing the fatigue failure of the construct by reducing the stress of the cerclage wire under tension load or distributing the tension load in the at-risk area of implant failure, such as the twisted knot (6–8), from the welding joint area between the double loop and resulting in the breakage of wire, as depicted in Tables 2 and 3. These results also confirmed that the fixation stability of cerclage wire fixation mainly depends on the wire tension and the twisting technique (6, 9). In addition, the findings on the single loop with welding on the twisted knot showed that the additional fixation stability from laser welding should be related to the welding area (0.74 mm in the single-loop group). Therefore, the application of laser welding in orthopedic fixation should be appropriately designed with the welding area, which is important for long-term durability.

Our hemolysis test also reported non-hemolytic results according to ASTM F756-13, ISO 10993-4, and ISO 10993-12 protocols, which represents that laser welding is not an acute *in vitro* hemolytic adverse event for use in contact with blood. However, other adverse biological effects—such as the incompetent welding process, the discontinued micro-surface of stainless steel, and the long-term release of metallic ions—are not assessed. Although the thermal effect is not evaluated in our study, Quazi et al. reported that the temperature alterations during welding are below the allowable limit of 5.5°C, encouraging the researchers to employ laser welding in *in vivo* studies, thus accounting for an immediate restoration of dental devices (11). Due to the small welding area, the surrounding tissues can be protected and cooled with saline solution during the intraoperative welding process. Moreover, this publication is the first that uses laser welding in orthopedic treatment. However, a small number of previous studies do describe laser welding applications. Hart and Wilson studied the biomechanics of using a filler wire in the laser welding of titanium prostheses to improve flexural strength and fatigue resistance (17). Ortorp and Jemt reported a 100% success rate with excellent overall long-term results of laser-welded titanium frameworks in mandible implantation (18). Fornaini et al. studied pig jaws (*ex vivo*) and found that laser welding could connect titanium implant abutments without the risk of thermal increase into the bone and with good results in terms of mechanical strength (19). Moreover, Fornaini et al. also revealed a case report of intraoral laser welding in a dental prosthesis; the results showed that it was a safe and effective procedure without any complications (20). On the basis of this study, we believe that the laser welding concept could safely be applied to cerclage wire fixation.

Our study has encountered some limitations. First, inherent to any biomechanical study is the difficulty in translation to clinical practice, which may be a combination of multi-loading directions, but we examined only one mode of loading. Consequently, while many previous clinical studies use welding materials in dentist surgery (21–23), further clinical studies and long-term results of using welding materials in orthopedic surgery are needed. Second, we emphasize only the mechanical strength and hemocompatibility of laser welding technology, an emphasis that does not purport to address all safety concerns. Nevertheless, other biocompatible testing, such as thermal necrosis and corrosion testing, should be clarified before human use. Third, the location, character, and magnitude of the welded area are created based on the feasibility and previous trial of examiners. Therefore, additional experiments should be performed for optimal outcomes. According to a different laser parameter between single- and double-loop specimens, a limited comparison between the groups is expected.

## 5. Conclusion

Laser welding significantly increases the biomechanical strength of double cerclage wire fixation and provides secure hemocompatibility. However, further biocompatible and clinical studies are still recommended.

## Data Availability

The original contributions presented in the study are included in the article, further inquiries can be directed to the corresponding author.
